# Sleep Quality Aspects in Post-COVID-19 Patients

**DOI:** 10.3390/jpm13071125

**Published:** 2023-07-11

**Authors:** Ioana Munteanu, Monica Marc, Constantin Gheorghevici, George Alexandru Diaconu, Nicolae Feraru, Dragos Sion, Roxana Maria Nemes, Beatrice Mahler

**Affiliations:** 1 Faculty of Medicine, Titu Maiorescu University, 040441 Bucharest, Romania; 2 “Marius Nasta” Institute of Pneumophtisyiology, 050159 Bucharest, Romania; 3 Department of Pneumology, “Victor Babes” University of Medicine and Pharmacy, 300041 Timisoara, Romania

**Keywords:** COVID-19, Sleep Apnea Syndrome, Pittsburgh Sleep Quality Index (PSQI)

## Abstract

In this observational cohort study, sleep quality in post-COVID-19 patients was assessed using the Pittsburgh Sleep Quality Index (PSQI) questionnaire. This study aimed to examine aspects of sleep quality in patients who have undergone SARS-CoV-2 infection and if there is a pattern of progression or regression over time (6 months). We also observed and analyzed the results in order to identify any possible links between the severity of COVID-19 and sleep quality as measured by the PSQI questionnaire. The study group consisted of 65 adult patients with confirmed SARS-CoV-2 infection who were referred to a pulmonologist for evaluation. Sleep quality was impacted at a high rate in post-COVID-19 patients, quantified by a PSQI score ≥5. Out of 65 patients, 51% of them had scores greater than or equal to 5. Sleep was subjectively reported as unsatisfactory predominantly in mild and moderate COVID-19 patients. According to the PSQI score and a 6-month follow-up, many patients presented persistency in poor sleep quality. Investigation and individualized treatment of sleep disorders in post-COVID-19 patients should be part of the routine pneumological control, as improvement in sleep quality has an impact not only on the health but also on the psychological state of patients. Educating patients about the importance of sleep and sleep quality impairment should be a primary concern.

## 1. Introduction

In 2019, the SARS-CoV-2 infection spread at an alarming rate, quickly becoming a pandemic, causing serious concerns and global psychological stress. The consequences of the pandemic itself and the countermeasures adopted to control infections and deaths have been associated with increased anxiety, stress, depression, and decreased sleep quality. Depression and a widespread increase in anxiety are risk factors associated with sleep disorders. Excessive worry about the evolution of the pandemic, one’s own health or the health of loved ones, financial issues, in addition to social restrictions, contribute to sleep impairment.

Sleep is an important biological mechanism for maintaining internal homeostasis, being an essential component for preserving health, physical, mental, social, spiritual functioning, and overall quality of life and plays a key regulatory role in the immune system. Reduced sleep quality can compromise immunity, increasing the likelihood of disease [1].

The specific pathophysiological mechanism leading to poor sleep quality post-COVID-19 has not been precisely established. It is thought that persistent viral infection indicated by the persistence of SARS-CoV-2 RNA for up to 230 days after symptom onset affects the hypothalamus and brainstem and may disrupt the sleep–wake cycle. This would lead to insomnia and poor sleep quality. Another mechanism would be persistent inflammation post-COVID-19, which can alter sleep by imbalancing the REM vs. non-REM sleep ratio [2].

In meta-analytic studies, the prevalence of sleep disorders associated with the COVID-19 pandemic is about 40%, consistently associated with psychological distress. In 2020, the share of sleep disorders per total was 36%, increasing steadily until 2021, reporting 47%. Patients infected with SARS-CoV-2 have a high predisposition for sleep disturbance due to the underlying symptoms of the disease, such as cough, fever, dyspnea, pain, and probably side effects of medications used [3,4].

Sleep disorders associated with COVID-19 are currently defined as “coronasomnia.” Coronasomnia is manifested by difficulty initiating/maintaining sleep or waking up early, fragmented sleep, paradoxical insomnia, and excessive diurnal sleepiness. Coronasomnia is caused by anxiety and stress, depression, changes in working hours, and prolonged use of social platforms. Studies on insomnia and COVID-19 show significant associations between them in affected individuals. The prevalence of insomnia symptoms in people with COVID-19 was 36 to 88%, which is significantly higher than the estimated insomnia prevalence of 10 to 40% in the general population [5].

This study aims to examine aspects of sleep quality in patients who have undergone SARS-CoV-2 infection and if there is a pattern of progression or regression over time (6-month follow-up). We also observed and analyzed the results in order to identify any possible links between the severity of COVID-19 and sleep quality as measured by the PSQI questionnaire. This paper presents a Romanian pulmonologist’s expertise with patients who have experienced SARS-CoV-2 infection and are suffering from sleep impairment.

## 2. Materials and Methods

The objective of the study was to assess sleep quality in post-COVID-19 patients. Follow-up after 6 months.

The study took place in the “Marius Nasta” Institute of Pneumophtisyology, Bucharest, Romania, from January 2022 to December 2022.

An observational cohort study was conducted in which participants completed the Pittsburgh Sleep Quality Index (PSQI) questionnaire for 15 min at the first visit and at 6 months thereafter.

The PSQI questionnaire is a valid and reliable international scale to assess subjective sleep quality. The total score is from 0 to 14, and scores equal to or greater than 5 indicate disturbed sleep quality. In addition to the PSQI questionnaire, the severity of COVID-19 that patients suffered was also scored as follows: mild form—those without oxygen therapy requirement; moderate form—with low-flow oxygen therapy requirement (<5 L/min); and severe form—with high-demand oxygen therapy requirement (>5 L/min).

Inclusion criteria: We included in this study any adult patient who presented to a pulmonology consultation, during day hospitalization or continuous hospitalization, who had a history of SARS-CoV-2 infection confirmed by a positive rapid antigen test (nasal swab/saliva), RT-PCR test, or any government document that certified the infection, including SMS from CORONAFORMS (Romanian COVID-19 platform), regardless of the date of infection, with their consent.

IBM SPSS 20 software was used where a database of the patient group was created, and subsequently statistical data were calculated using this software.

Exclusion criteria: Any patient who denied a history of SARS-CoV-2 infection, did not consent to participate in the study, refused to communicate the required information, or did not communicate it completely was excluded from the study group.

Ethics Committee Approval Request No. 28301/07.12.2021. Approved by the Ethics Committee on 13 December 2021.

## 3. Results

### 3.1. Severity

We included 65 patients with a history of confirmed SARS-CoV-2 infection. There were 41 patients that experienced mild COVID-19 (63%), 12 patients with moderate disesase (18.5%), and 12 with severe disease (18.5%) (Table 1).

### 3.2. Demographics

**Figure 1:**
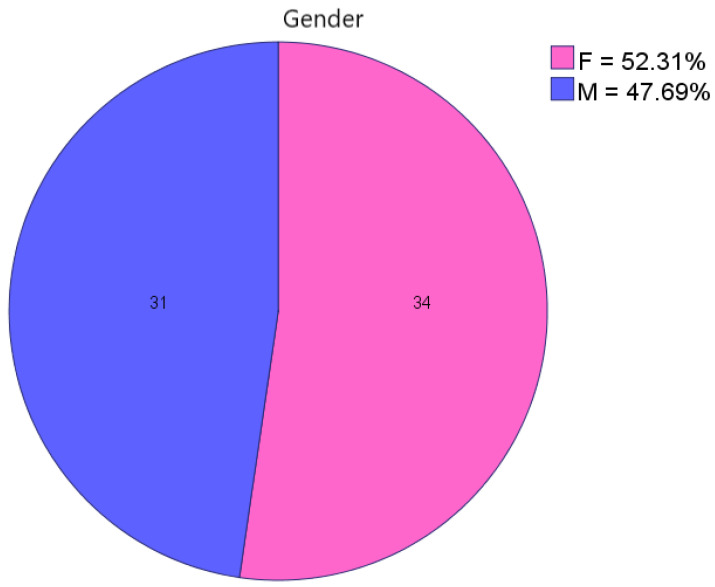
Gender distribution by number and percentage.

The gender distribution was approximately equal, with a slight predominance of females. A total of 31 male patients (48%) and 34 female patients (52%) participated in the study (Figure 1).

**Figure 2:**
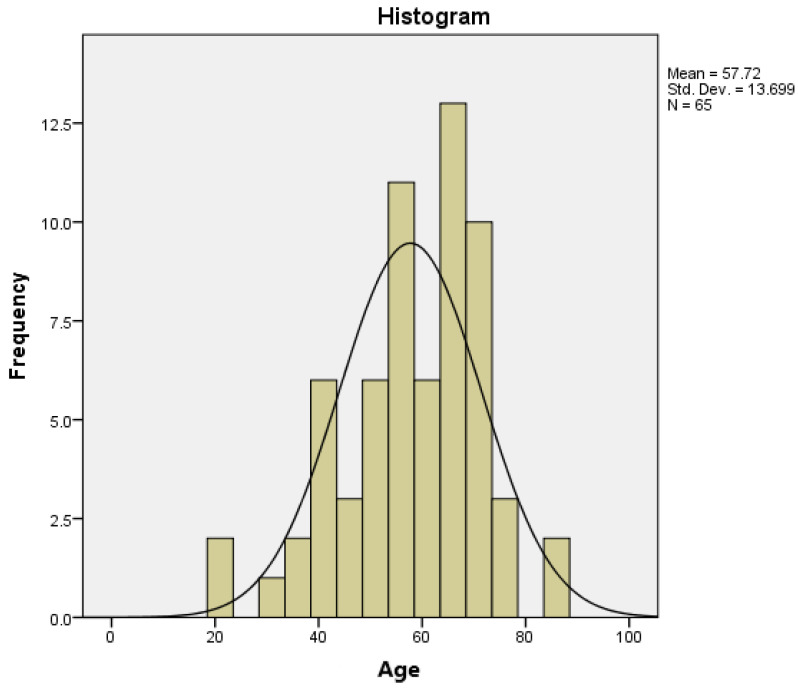
Age histogram showing normal curve.

Patients ranged in age from 21 years to 85 years, with a median age of 57.7 years (Figure 2).

### 3.3. First Visit Results

Of the total 65 patients who completed the PSQI questionnaire, 33 of them (51%) had scores equal to or greater than 5, indicating disturbed sleep quality (Table 2).

In terms of disease severity, 51% of those with mild disease, 58% of those with moderate disease, and 42% of those with severe forms had a score greater than or equal to 5 (Table 2).

**Figure 3:**
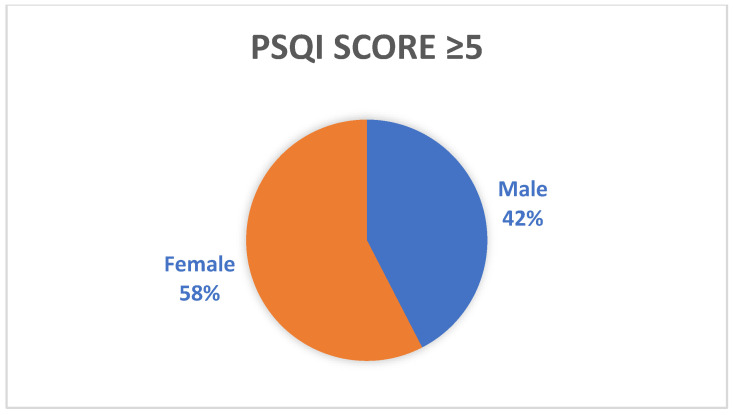
Sex distribution in patients with impacted sleep quality.

By gender, there was a higher prevalence of sleep disorders among females. (Figure 3).

**Figure 4:**
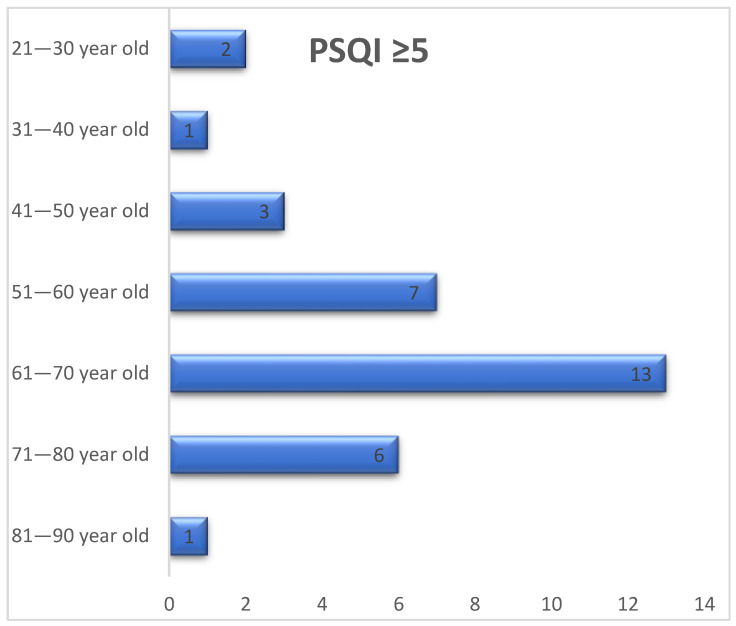
Number of patients with PSQI score equal to or higher than 5, by age groups.

More than three quarters of the cases with a PSQI score ≥ 5 occurred in patients aged 51–80 years, with the percentage being about 79% between this age range. Sleep quality was affected in a higher percentage in those in their 6th decade of life (Figure 4).

Of the patients surveyed, those with moderate forms of COVID-19 subjectively rated their sleep quality as partially bad in a high percentage (28.6%), compared to those with mild forms (9.1%) and those with severe forms (9.1%). Sleep duration was also shorter, and sleep latency was higher in this group of patients (Table 3).

### 3.4. Second Visit (6-Month Follow-Up)

At the 6-month reassessment, 38 of the 65 initially surveyed patients came for the follow-up examination.

In relation to the severity of COVID-19, 58% of those with mild disease had a PSQI score greater than or equal to 5, and 67% of those with moderate disease and 50% of those with severe disease also scored equal to or greater than 5 (Table 4).

Patients subjectively rated their sleep quality as partly good or very good in a high percentage of instances, with only 12.9% of those with mild COVID-19 reporting their sleep quality as partly bad. In contrast, sleep latency remained high in one third of those with moderate forms of COVID-19. Sleep duration was relatively equal in all patient groups, approximately 6 h 30 min. (Table 5).

## 4. Discussion

The data analyzed by us raise awareness about the high percentage of the population experiencing sleep problems.

Mekhael et al. conducted a prospective observational study on two groups of patients, a group of post-COVID-19 patients and a group of patients without prior SARS-CoV-2 infection. They evaluated the long-term effects of COVID-19 on sleep quality using biometric data-monitoring strips and concluded that regardless of demographic background and symptom severity, patients with SARS-CoV-2 infection had altered sleep architecture, characterized by decreased total sleep and deep sleep times compared with other patients without SARS-CoV-2 infection [6].

Post-COVID-19-altered sleep quality was frequently reported in the recovery period, negatively impacting physical and mental health [7]. This aspect was present in our studied population as well, although we do not have information regarding patient baseline sleep quality.

Sleep quality was impacted in a high percentage of post-COVID-19 patients, quantified by a PSQI score ≥ 5. About 51% of them had scores greater than or equal to 5. In meta-analytic studies, the prevalence of sleep disorders associated with the COVID-19 pandemic globally is approximately 40% [3,4], although it is difficulty to say if these numbers are a cause of the disease itself or the pandemic-adjacent restrictions, such as lockdowns, lack of physical contact, or changing the job venue (from an office to a room inside the house).

By gender, a slightly higher prevalence of sleep disorders was observed among females. Sleep quality was impaired at a higher rate in those aged 51–80 years, but the study group had a median age of 57 years. In a meta-analysis, it was reported that age and gender had no influence on the estimated prevalence of sleep disturbance [3].

According to the PSQI score, sleep disturbances persisted at 6 months in many patients. Because not all patients presented for reassessment, we cannot estimate percentage-wise whether sleep quality worsened at 6 months. Scientific evidence suggests that sleep disorders associated with COVID-19 may become chronic [5,8].

Other evidence shows that dysregulation of the post-COVID-19 immune response can cause sleep dysfunction, i.e., mitochondrial destruction under post COVID-19 conditions can release a wide range of molecules with an activating effect on the inflammatory response. This process can damage neurons in the hypothalamus and brainstem area affecting circadian rhythm [2]. In our data, 22 out of 38 patients (57.89%) scored at least 5 points in the PSQI questionnaire during their 6-month follow-up consult, suggesting the chronicity of the condition.

A study by Morin et al. involving 594 adult patients compared both the prevalence of insomnia symptoms and the diagnosis established before the declaration of the COVID-19 pandemic (2018) and in the first wave (2020). Data on incidence, prevalence, and persistence of insomnia, anxiety, depressive symptoms, anxiety, and stress levels were included. After analyzing data from the two periods, the authors conclude a 26.7% increase in insomnia rates. Also, 32.6% of patients who were considered good sleepers before the pandemic developed insomnia during the COVID-19 period [9]. In our experience, 40/65 patients (61.5%) perceived their sleep as “poor-quality”. We do not have the necessary data to explore their subjective sleep quality before the SARS-CoV-2 infection.

Similarly, Meaklim, Hailey et al. examined levels of stress, anxiety, and depression in patients diagnosed with insomnia before and after the pandemic and in those without sleep problems. The analysis was based on a questionnaire, and the study included 2724 respondents from 67 countries. Patients who were diagnosed with insomnia after the onset of the pandemic had higher levels of anxiety, stress, and depression compared to those who were insomniacs before the pandemic or those who did not have a diagnosis of insomnia [10].

Zhao, Yongzhi et al. carried out a similar study by administering a series of questionnaires to medical staff in the First People’s Hospital of Kashi, Xinjiang during the second wave of COVID-19. The prevalence of insomnia among the respondents was 41.1%. High levels of stress, anxiety, and depression were also recorded among the medical staff assessed. The authors concluded that worries about the risk of developing disease, disease research, and neurotic personality were associated with the occurrence of mental health problems. Conversely, high educational attainment, well-represented social support, and extroverted personality were negatively associated with mental health problems [11].

Cybulska, Anna Maria et al. investigated risk factors associated with insomnia and aggression in healthcare workers. The Buss–Perry Aggression Questionnaire, Athens Insomnia Scale, Pittsburgh Sleep Quality Index, and a self-administered questionnaire were used on 264 healthworkers. The result of the questionnaires was that 81% of the respondents suffered from insomnia and 78% had poor sleep quality, a higher percentage than in our studied population. Marital status, education level, and working with COVID-19 patients were contributing factors to insomnia levels. Older age was correlated with higher levels of verbal aggression and anger among healthworkers. From a statistical point of view, the Buss–Perry Aggression Questionnaire and the Pittsburgh Sleep Quality Index were positively correlated [12].

A meta-analysis was conducted on the mental health of key populations in Africa. Chen, Jiyao et al. reviewed 28 studies and 32 independent samples from 12 African countries, totaling approximately 15,000 participants. Across the continent, the prevalence of insomnia was 28%. North Africa recorded higher values of insomnia (31%) than Sub-Saharan Africa (24%). Similarly, the North African population recorded higher values of anxiety and depression than the Sub-Saharan region [13]. It seems that the African population suffers more from depression than from sleep and anxiety disorders, but all these are interlinked pathologies.

Pudlo et al. conducted a prospective study of 200 patients with SARS-CoV-2 infection to assess the prevalence of insomnia in the early post-COVID-19 period. They concluded that the prevalence of insomnia in the early post-COVID19 recovery period was quite high, with one in five having the clinical criteria for the diagnosis. Another important finding was that persistent COVID-19 symptoms were positively correlated with the development of insomnia to a greater extent than the number of symptoms observed in the acute phase of the disease [14]. In our study, we did not include information regarding the persistence of COVID-19 symptoms to verify if there are links between “Long COVID” syndrome and altered sleep quality.

The comparison between PSQI scores at T0 and T6 (months) is a new contribution and shows the need for new and larger-scale studies to draw relevant conclusions about the chronicity of sleep impairment by the COVID-19 pandemic.

## 5. Conclusions

The COVID-19 pandemic has had a negative impact on sleep quality and therefore on quality of life. Impaired sleep quality was observed in patients at the first visit and at the 6-month reassessment, so it can be alleged that sleep disturbances persist over time in post-COVID-19 patients.

We did not proportionally correlate the severity of the disease with the PSQI score; therefore, other factors might play a role in developing sleep disturbances.

This study shows a disturbingly high number of patients who perceive their sleep as unrefreshing or of poor quality. More and larger studies, which include pre-COVID-19 baseline parameters and other environmental factors, are needed on this topic to investigate whether these problems occur to the same extent in the non-COVID-19 population.

Investigation and individualized treatment of sleep disorders in post COVID-19 patients should be part of the routine pneumological control, as improvement in sleep quality has an impact not only on the health but also on the psychological state of patients.

Sleep disorders associated with COVID-19 may become chronic and will continue to be of concern to somnologists even after the pandemic is over.

Educating patients about the importance of sleep and the risk of decreased sleep quality during this or future pandemics should be a primary concern.

## Figures and Tables

**Table 1:** Prevalence of disease severity in the study population.

**Patients**	**COVID-19 Severity**		
	**Mild**	**Moderate**	**Severe**
	41	12	12
	Total: 65 pacients		

**Table 2:** Crosstabulation of PSQI score and COVID-19 severity during the first consult.

COVID-19 Severity					Total
		Mild	Moderate	Severe	
	0	1	0	1	2
	1	1	0	2	3
	2	8	3	0	11
	3	4	0	2	6
	4	6	2	2	10
	5	5	1	2	8
**PSQI SCORE**	6	4	3	1	8
	7	3	0	1	4
	8	3	1	0	4
	9	2	0	1	3
	10	1	1	0	2
	11	1	0	0	1
	12	1	0	0	1
	14	1	0	0	1
	15	0	1	0	1
**Total**		41	12	12	65

**Table 3:** Crosstabulation of PSQI items and COVID-19 severity during the first visit. (G = good; B = bad).

Disease Severity	Sleep Duration	Sleep Latency (in Minutes)			Subjective Assessment of Sleep Quality				Sleep Induction Medication		Enthusiasm for Daily Activities		
		≤15	15–30	≥30	Partially Good	Partially Bad	Very Good	Very Bad	YES	NO	YES	NO	Partially
Mild	7 ± 1.8 h	54.1%	37.8%	8.1%	58.3%	9.1%	27.3%	0%	11.1%	88.9%	45.9%	21.9%	32.4%
Moderate	6 ± 1.6 h	42.9%	28.6%	28.6%	71.4%	28.6%	0%	0%	14.3%	86%	85.7%	0%	14.3%
Severe	8 ± 1.9 h	54.6%	36.4%	9.1%	63.6%	9.1%	27.3%	0%	9.1%	90%	63.6%	9.1%	27.3%

**Table 4:** Crosstabulation of PSQI score and COVID-19 severity after 6 months.

COVID-19 Severity					Total
		**Mild**	**Moderate**	**Severe**	
	1	3	0	0	3
	2	2	1	2	5
	3	5	0	0	5
	4	3	0	0	3
	5	9	0	2	11
**PSQI SCORE**	6	1	0	0	1
	7	2	1	0	3
	8	2	0	0	2
	9	1	0	0	1
	11	0	1	0	1
	12	1	0	0	1
	14	1	0	0	1
	15	1	0	0	1
**Total**		31	3	4	38

**Table 5:** Crosstabulation of PSQI items and COVID-19 severity after 6 months. (G = good; B = bad).

Disease Severity	Sleep Duration	Sleep Latency (in Minutes)			Subjective Assessment of Sleep Quality				Sleep Induction Medication		Enthusiasm for Daily Activities		
		≤15	15–30	≥30	Partially Good	Partially Bad	Very Good	Very Bad	YES	NO	YES	NO	Partially
Mild	6.64 h	58.1%	32.3%	9.7%	67.7%	12.9%	19.7%	0%	9.7%	90.3%	61.3%	3.2%	35.5%
Moderate	6.5 h	33.3%	33.3%	33.3%	100%	0%	0%	0%	0%	100%	66.7%	0%	33.3%
Severe	6.75 h	75%	25%	0%	50%	0%	50%	0%	0%	100%	75%	0%	25%

## Data Availability

Data used to support the findings of this study are available from the corresponding author upon request.
